# Altered *PCA3* and *TMPRSS2-ERG* expression in histologically benign regions of cancerous prostates: a systematic, quantitative mRNA analysis in five prostates

**DOI:** 10.1186/s12894-015-0077-7

**Published:** 2015-08-21

**Authors:** Riina-Minna Väänänen, Natalia Tong Ochoa, Peter J. Boström, Pekka Taimen, Kim Pettersson

**Affiliations:** Department of Biotechnology, University of Turku, Turku, Finland; Department of Urology, Turku University Hospital, Turku, Finland; Department of Pathology, University of Turku and Turku University Hospital, Turku, Finland

**Keywords:** Prostate, Cancer, Biomarker, RNA, Reverse transcriptase polymerase chain reaction

## Abstract

**Background:**

*PCA3* and *TMPRSS2-ERG* are commonly overexpressed biomarkers in prostate cancer, but reports have emerged demonstrating altered expression also in areas outside the tumour foci in cancerous prostates. Our aim was to measure *PCA3* and *TMPRSS2-ERG* expression systematically in all regions of prostate cross-sections, matching the data to corresponding tissue morphology.

**Methods:**

*TMPRSS2-ERG* and *PCA3* mRNA levels were measured with quantitative reverse-transcription PCR assays in 270 samples from cross-sections of five radical prostatectomy specimens. ERG expression was examined by immunohistochemistry.

**Results:**

*TMPRSS2-ERG* mRNAs were detected in three patients and in 15 tissue samples in total. These included two carcinoma samples and 13 histologically benign samples, eight of which were located next to malignant tumours or PIN (prostatic intraepithelial neoplasia) lesions and five of which did not reside in the vicinity of any evident carcinoma foci. ERG protein expression was limited to areas of *TMPRSS2-ERG* mRNA expression, but did not identify all of them. *PCA3* expression was detected in all five cross-sections, with statistically significant, three-fold higher expression in carcinoma regions.

**Conclusions:**

*TMPRSS2-ERG* expression was detected in carcinoma foci, regions next to them, and in samples not adjacent to carcinoma foci. Claimed as a cancer-associated phenomenon, this fusion gene measurement could, if validated with a larger cohort, be utilized as an addition to histological analysis to predict current or future cancer risk in men with negative biopsies. Molecular changes outside the carcinoma foci are also indicated for *PCA3*, as its expression was only moderately increased in the carcinoma regions.

## Background

The proposed idea of field cancerization – phenomenon first described by Slaughter and colleagues in 1953 [1] – comprises the assumption that in cancers, originally a larger area of the tissue than merely the tumour focus can be changed due to inherent mutations or environmental factors. The hypothesis has been supported by several studies reporting molecular level alterations not only in the carcinoma tissue of an organ containing a malignant tumour, but also in the area outside the cancerous region [2, 3]. Both aberrant protein expression and mRNA transcript levels have been described [4]. Besides providing an interesting perspective to understanding carcinogenesis, detectable consequences of the field effect phenomenon could be used to supplement diagnostics.

Prostate cancer is a disease with a growing incidence and a heterogeneous nature, and the heterogeneity of the disease has made diagnostics and prognostics challenging. The currently used biomarker, PSA (prostate specific antigen) measured from blood, cannot reliably confirm the presence of cancer in the prostate since increased levels are frequently found also in other prostatic diseases such as hyperplasia and prostatitis. Present routine to establish the prostate cancer diagnosis is based on the histological examination of core needle biopsies that are taken with trans-rectal ultrasound guidance. However, the biopsy cores represent a random sampling of the overall tumour load regarding the aiming of the biopsy needle to the estimated carcinoma location. A histologically benign biopsy result leads to a negative cancer diagnosis, but based on the previous reports on molecular level alterations in cancer-adjacent tissues, it may be premature in determining the status of the patient.

Alterations in the expression of prostate cancer marker candidate genes *TMPRSS2-ERG* (transmembrane protease, serine 2; ETV-related gene) and *PCA3* (prostate cancer antigen 3) have previously been detected by us and others [5–9]. The changes were specifically seen in the histologically benign areas of cancerous prostates but not in similar areas of prostates that were free of clinical cancer. This study was designed to systematically locate the regions of differential expression of these genes in single cross-sections of five cancerous prostates and evaluate whether the location of the carcinoma was associated with *TMPRSS2-ERG* or *PCA3* mRNA levels or ERG protein expression.

## Methods

### Sample collection

**Fig. 1:**
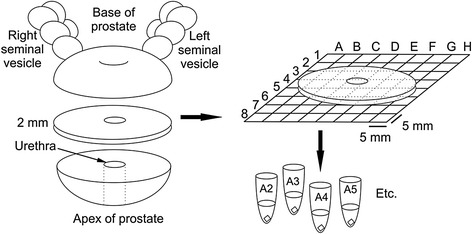
Flowchart of the sample collection protocol for mRNA experiments. A horizontal cross-section slice of 2 mm in thickness was cut from the middle of each prostate and laid flat on a cutting plate while recording the original orientation of the slice in the organ. The slice was further cut into 5x5 mm pieces according to a grid and each sample was stored separately in an RNA stabilizer solution

To measure the mRNA expression of the target genes by quantitative reverse-transcription PCR (qRT-PCR) in prostate tissue, prostate cross-sections covering the entire organ were obtained fresh from five prostates (hereafter referred to as prostates A, B, C, D, and E) from men undergoing robotic assisted laparoscopic radical prostatectomies due to prostate cancer at Turku University Hospital, Turku, Finland in June–September 2013. Five consecutive patients with previous diagnosis of prostatic adenocarcinoma in transrectal biopsies were enrolled in the study. Patients with diagnosed adenocarcinoma in both lobes and patients with clinical suspicion of multifocal or large tumour were excluded from the study. The sample collection protocol is depicted in Fig. 1. Briefly, a horizontal tissue slice of 2 mm in thickness was removed from each prostate and further cut into 5x5x2 mm pieces systematically with sterile blades, avoiding cross-contamination between pieces. A Styrofoam plate with a 5x5 mm grid was used to record the two-dimensional location of each piece, resulting in a unique coordinate code for each piece of tissue. Depending on the size of the organ, this procedure yielded 48 individual samples for prostate A, 62 samples for prostate B, 44 samples for prostate C, 55 samples for prostate D, and 61 samples for prostate E. All pieces were stored separately in RNA*later* RNA Stabilization Reagent (Qiagen, Hilden, Germany) at −20 °C.

Tissue immediately adjacent to the tissue cross-section used in mRNA measurements was fixed in formalin and embedded in macro paraffin blocks (FFPE) to enable examination of tissue morphology. Sections were cut directly from the superior and inferior side of the cross-section used in mRNA measurements, stained with hematoxylin and eosin (HE), and inspected for cancer foci and prostatic epithelial neoplasia (PIN) lesions by an experienced uro-pathologist. The locations of carcinoma areas and PIN lesions were marked and the slides were scanned into digital images. All five prostate cross-sections contained cancerous areas and cross-sections B and C contained also PIN lesions.

The study protocol was approved by the Ethics Committee of the Hospital District of Southwest Finland and it was in accordance with the Helsinki Declaration of 1975, as revised in 1996, with written informed consent obtained from each participant.

### Real-time PCR for *PCA3* and *TMPRSS2-ERG* mRNAs

RNA extraction and reverse transcription were performed with RNeasy Mini kit (Qiagen, Germany) and High Capacity cDNA Archive kit (Applied Biosystems, USA) according to manufacturer’s instructions and as described previously [10]. Artificial internal standard RNA was added to each sample at the beginning of RNA extraction process, after cell lysis [11].

Quantitative real-time PCR assays using a closed-tube concept [12] and time-resolved fluorometry-based detection were performed as described previously [9, 10] to measure *PCA3* and *TMPRSS2-ERG* mRNA levels in each tissue piece. Levels of *KLK3* (kallikrein-related peptidase 3, gene encoding PSA) mRNA and internal standard RNA were also measured for control and normalization purposes [11–14]. The oligonucleotide sequences are presented in Table 1. External DNA standards (Table 2) were used to form a standard curve and the lowest dilutions equal the limits of detection for the particular assays.

**Table 1:** Sequences of the primers and probes used in this study

Oligonucleotide	Sequence (5’- > 3’)	GenBank database sequence number	Position in sequence
*KLK3*			
5’ primer	TGA ACC AGA GGA GTT CTT GAC	X05332	523–543
3’ primer	CCC AGA ATC ACC CGA GCA G	X05332	667–685
reporter probe	CCT TCT GAG GGT GAA CTT GCG C	X05332	596–617
quencher probe	AAT CAC CCT CAG AAG G	X05332	600–601, 604–617
mmPSA			
5’ primer	TGA ACC AGA GGA GTT CTT GCA	X05332	523–543
3’ primer	CCC AGA ATC ACC CGA GCG A	X05332	667–685
reporter probe	CCT TCT GAG GGT GAT TGC GCA C	X05332	594–601, 604–617
quencher probe	AAT CAC CCT CAG AAG G	X05332	600–601, 604–617
*PCA3*			
5’ primer	GGT GGG AAG GAC CTG ATG ATA C	AF103907	95–116
3’ primer	GGG CGA GGC TCA TCG AT	AF103907	505–521
reporter probe	AGA AAT GCC CGG CCG CCA TC	AF103907	478–497
quencher probe	CCG GGC ATT TCT	AF103907	478–489
*TMPRSS2-ERG* III			
5’ primer	TAG GCG CGA GCT AAG CAG GAG	NM_005656.3	4–24
3’ primer	GTA GGC ACA CTC AAA CAA CGA CTG G	NM_004449.4	338–362
reporter probe	AGC GCG GCA GGA AGC CTT ATC AGT T	NM_005656.3 and NM_004449.4	57–64 and 310–326
quencher probe	TTC CTG CCG CGC T	NM_005656.3 and NM_004449.4	57–64 and 310–314
*TMPRSS2-ERG* VI			
5’ primer	CGG CAG GTC ATA TTG AAC ATT CC	NM_005656.3	73–95
3’ primer	GCA CAC TCA AAC AAC GAC TGG	NM_004449.4	338–358
reporter probe	CTT TGA ACT CAG AAG CCT TAT CAG TTG TGA	NM_005656.3 and NM_004449.4	139–149 and 312–330
quencher probe	GGC TTC TGA GTT CAA AG	NM_005656.3 and NM_004449.4	139–149 and 312–317

Lanthanide chelates were attached to the 5’ ends of the reporter probes via an amino group to enable signal measurement with time-resolved fluorescence and phosphate groups to the 3’ ends to prevent them from functioning as starting points for DNA synthesis. Quencher molecules were attached to the 3’ ends of the quencher probes. The oligonucleotide sequences have been previously published for *KLK3* [12, 13], mmPSA [11–13], *PCA3* [10, 20], *TMPRSS2-ERG* III [9, 21, 22], and *TMPRSS2-ERG* VI [9] assays

**Table 2:** Dilutions of external DNA standards used in the real-time PCR assays

	Range (molecules per mL of template)		
Target RNA	Lowest concentration	Highest concentration	Total number of points on standard curve
*KLK3*	2.5 × 10^3^	2 × 10^11^	8
*PCA3*	1.3 × 10^3^	2.5 × 10^11^	7
*TMPRSS2-ERG* III	5 × 10^3^	5 × 10^7^	4
*TMPRSS2-ERG* VI	2 × 10^4^	2 × 10^8^	4

### Immunohistochemistry

For immunohistochemistry (IHC) experiments, sections of 3–4 μm were cut from the FFPE tissue macro blocks immediately from the superior and inferior side of the HE-stained sections. The sections were pretreated in xylene and ethanol, and the heat-induced antigen retrieval was performed in a microwave oven using Target Retrieval Solution (Dako) followed by cooling at RT. Staining was performed by incubating the slides for one hour at RT in a humid chamber with rabbit monoclonal ERG antibody (clone EPR3864; Epitomics) that was used in 1:250 dilution. EnVision™ + Dual Link System-HRP (Dako) was used as the secondary antibody with a 30-min incubation at RT. The staining was visualized by incubation in DAB+ Chromogen solution (Dako) for 10 min at RT. After counterstaining with hematoxylin, dehydration, and treatment by xylene, the slides were analysed by an experienced uro-pathologist. The vascular endothelial cells served as positive controls for the staining with ERG antibody and cells of the benign glands as the negative control.

### Data analysis

The specific locations of tissue were translated to match samples used for mRNA measurements by dividing the digital images of HE-stained tissue slides into equal amount of regions. Thus, potential shrinkage of the tissue was accounted for on average. Each sample piece was given coordinates on two axis (one giving values from A to K, and the other giving values from 1 to 10). Samples were classified into four categories based on morphology-based examination of the immediately adjacent HE-stained tissue. Sample was deemed as a carcinoma sample if one or both studied HE sections revealed adenocarcinoma at the same location, and PIN if one or both studied HE sections revealed PIN lesion. If both studied HE sections contained only histologically benign tissue in that area but the sample immediately next to the sample was classified as carcinoma or PIN sample, the sample was classified as “histologically benign tissue immediately adjacent to a carcinoma/PIN sample” (HBAC). If HE sections showed only histologically benign tissue in the sample and the samples next to it, sample was marked as histologically benign area (HB).

Samples were considered to contain measurable amounts of target mRNAs only when three PCR replicates gave a rise in fluorescence signals. Copy numbers were calculated as previously described [10], taking into account the potential RNA loss in extraction by using an internal RNA standard. For *TMPRSS2-ERG* mRNAs, samples that produced signal in only one or two out of the three PCR replicates were considered as samples where *TMPRSS2-ERG* mRNA expression was detectable but not quantifiable. Limits of detection for the real-time PCR assays are presented

Mann–Whitney *U* test was used to study associations between mRNA and protein expression of the target genes and the histology of the tissue (SPSS 20.0, IBM).

## Results

### Patient and tumour characteristics

The essential clinicopathological characteristics of the five cases are presented in Table 3. All cases were clinically and pathologically organ confined. Two patients (B and D) had 5-alpha-reductase inhibitor therapy and one patient (E) had a combination therapy of 5-alpha-reductase inhibitor and α_1_ receptor antagonist prior to surgery. No positive margins were detected and all patients achieved serum PSA of <0.1 ng/mL postoperatively. Using ultrasensitive PSA measurement, two patients had detectable postoperative serum PSA (0.004 and 0.026 ng/mL). During the follow-up of 8–11 months, none of the patients had experienced clinical or biochemical recurrences. Patients D and E had clearly one index tumour, patients A and C had two or more carcinoma foci (between 6 and 18 mm), and patient B had two small, well-differentiated carcinoma foci (both <10 mm).

**Table 3:** Characteristics of the five patients included in the study

Patient	Age	PSA (ng/mL)		Gleason sum	Tumour volume (%)
		preoperative	postoperative		
A	67	4.5	<0.003	3 + 4	10
B	59	9.2	<0.003	3 + 3	2
C	59	8.5	<0.003	3 + 4	5
D	67	16	0.026	4 + 3	8
E	66	18	0.004	4 + 3	15

Tumour volume in the whole prostate was estimated based on the macro sections taken every 5 mm and covering the whole organ

### *KLK3* mRNA expression

*KLK3* mRNA levels were measured from all samples for control purposes. There were no statistically significant differences in *KLK3* mRNA levels between carcinoma, PIN, HBAC, or HB samples (data not shown).

### *PCA3* mRNA expression

**Fig. 2:**
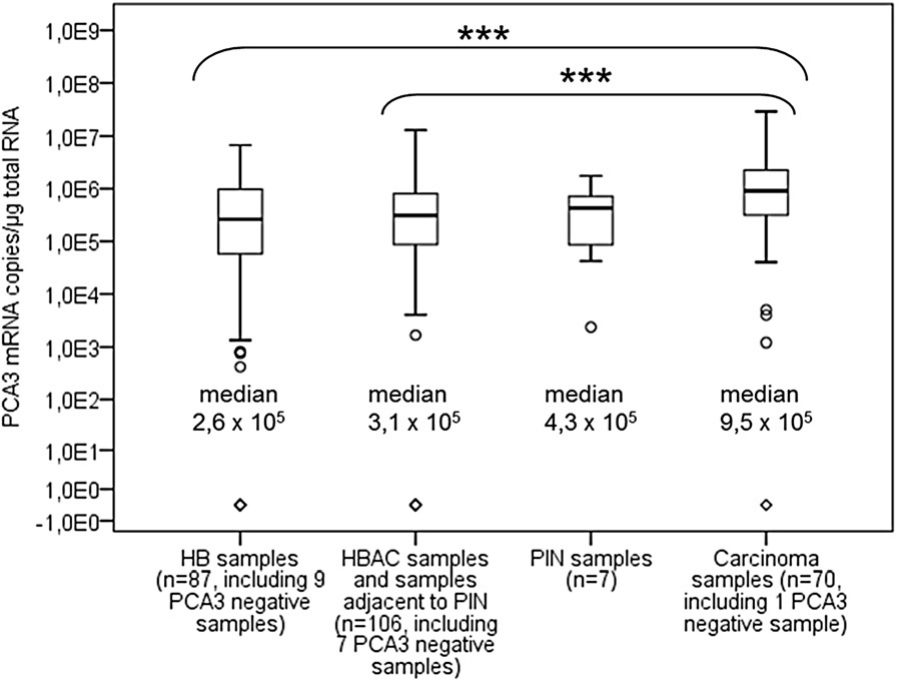
*PCA3* mRNA levels in tissue samples from the five studied prostates. The boxes contain interquartile ranges with median values shown as horizontal lines and the whiskers extending to minimum and maximum values. The statistical outliers are depicted with circles and negative samples with open diamonds. Statistically significant differences between the sample groups are marked with stars, and three stars denote a p value of less than 0.001. HB, histologically benign samples; HBAC, histologically benign samples adjacent to carcinoma; PIN, prostatic intraepithelial neoplasia

All five prostates showed *PCA3* mRNA expression. It was universally expressed in prostates A and B and in 80–96 % of samples of prostates C, D, and E. When all samples from the five prostates were combined, the median expression level was highest (9.54 × 10^5^ mRNA copies/μg total RNA) in the samples that were classified as carcinoma samples, second highest in PIN samples, third highest in samples adjacent to carcinoma or PIN samples, and lowest in HB samples (2.62 × 10^5^ mRNA copies/μg total RNA (Fig. 2). The statistically significant difference in median *PCA3* mRNA values between carcinoma samples and HB samples was 3.6-fold (*p* < 0.001). The difference between carcinoma samples and samples adjacent to carcinoma or PIN was 3.1-fold (*p* < 0.001).

When the five studied prostate cross-sections were looked at individually, the same trend of statistically significantly higher *PCA3* mRNA expression in carcinoma areas persisted only for prostate C, where the difference between medians of carcinoma and HB samples was 20-fold (*p* < 0.003), and for prostate D with a 5.2-fold difference (*p* < 0.001).

### *TMPRSS2-ERG* mRNA expression

**Fig. 3:**
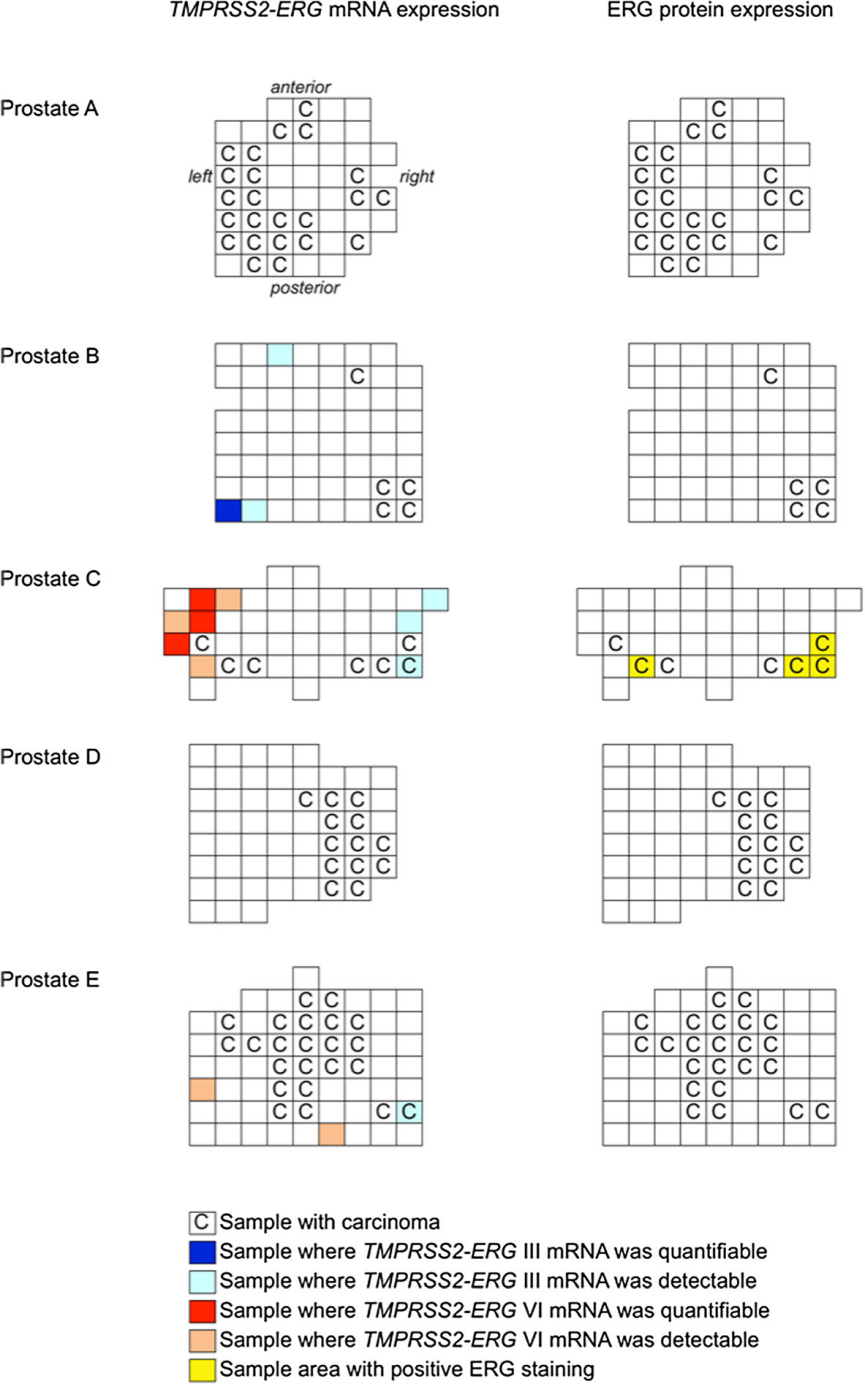
Location of detected *TMPRSS2-ERG* mRNA expression and ERG protein expression in relation to carcinoma areas. Morphologically determined carcinoma areas are marked with C in the five prostates. Dark blue boxes denote samples with quantifiable *TMPRSS2-ERG* III mRNA expression, and light blue boxes represent samples with detectable but not quantifiable *TMPRSS2-ERG* III mRNA expression. Red boxes denote samples with quantifiable *TMPRSS2-ERG* VI mRNA expression and light orange boxes represent detectable but not quantifiable *TMPRSS2-ERG* VI mRNA expression. Yellow boxes denote ERG protein expression. *TMPRSS2-ERG* mRNAs were found in prostates B, C, and E and ERG protein expression only in prostate C. None of the samples showed simultaneous expression of both *TMPRSS2-ERG* mRNA variants

*TMPRSS2-ERG* III mRNA was detected in 3 out of 5 prostates (B, C, and E) and *TMPRSS2-ERG* VI mRNA in 2 out of 5 prostates (prostates C and E). The samples containing detectable *TMPRSS2-ERG* expression and their location in relation to carcinoma areas are depicted in Fig. 3. Reliably quantifiable expression of *TMPRSS2-ERG* III or VI mRNAs was found in four histologically benign samples, two of which were HBAC samples. The third sample was located next to a PIN lesion and the fourth resided in an area that was regarded as histologically benign. In addition, detectable but not quantifiable expression of these mRNAs was found in one carcinoma focus of prostate C, one carcinoma sample of prostate E, in four HBAC samples, and in five HB samples. One of the HB samples was adjacent to a PIN area. None of the individual samples showed simultaneous expression of both *TMPRSS2-ERG* mRNA isoforms.

### ERG protein expression

**Fig. 4:**
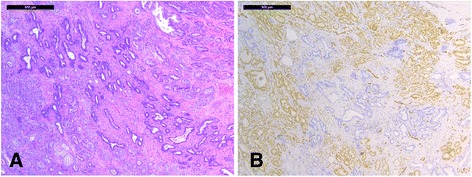
Histology of prostate C showing (**a**) HE staining and (**b**) immunohistochemical ERG staining from the same area. The nuclei of malignant glands stain positively for ERG suggesting that ERG is overexpressed due to *TMPRSS2-ERG* fusion. Scale bar 500 μm

IHC experiments detected positive ERG staining only in prostate C (Fig. 4). Based on the morphological analysis, all ERG positive areas contained carcinoma tissue. *TMPRSS2-ERG* mRNA was detectable in one out of the four individual tissue samples matching the ERG positive areas and the other three samples were located adjacent to *TMPRSS2-ERG* mRNA positive samples. One of the samples contained *TMPRSS2-ERG* III mRNA and the others *TMPRSS2-ERG* VI mRNA, but the amounts were not quantifiable in any of them. Areas in the same and other prostates containing either quantifiable or only detectable expression of *TMPRSS2-ERG* mRNAs were negative for ERG in IHC.

## Discussion

Field effect is a recognized phenomenon in several cancers, including prostate cancer. It suggests that larger areas of the tissue than just the histologically identifiable tumour regions are changed on the molecular level. We studied the extent and location of the mRNA expression of two suggested prostate cancer markers, *TMPRSS2-ERG* fusion gene and *PCA3*, in a systematic way in cross-sections of cancerous prostates containing both histologically benign and carcinoma areas.

In addition to overexpression in tumours, increased expression of *PCA3* has been described also in areas adjacent to carcinoma foci and the phenomenon was explained by a carcinogenic field effect [3]. The thus far reported findings of *TMPRSS2-ERG* in BPH (benign prostatic hyperplasia) tissue, or in a benign area from a cancerous prostate, have sometimes been speculated to result from small carcinoma foci that resided in sampled tissues and were unnoticed despite microscopic examination [5, 7]. Also the possibility of the samples containing precancerous tissue has been proposed [6, 7]. Our previous study, showing that 44 % of the histologically benign sampled areas of cancerous prostates contained *TMPRSS2-ERG* mRNA transcripts in contrast to none of the seven benign tissues of cancer-free prostates [9], led us to hypothesize on *TMPRSS2-ERG* expression not being limited to carcinoma foci in cancerous prostates.

The fusion gene mRNAs were detectable in two carcinoma samples in this study, and also in 13 histologically benign samples. However, eight of the *TMPRSS2-ERG* positive histologically benign samples resided immediately next to samples classified as carcinoma or PIN. Due to the sample collection set-up, those samples could also contain cancer cells originating from the adjacent sample areas as the protocol of matching tissue morphology with the location of samples used for mRNA measurements was not able to fully account for site-specific tissue shrinkage caused by the fixation of the tissue. Yet we detected *TMPRSS2-ERG* expression in five samples without evidence of carcinoma foci in the immediate vicinity. In contrary to our previous study [9], none of the individual samples in this limited cohort showed simultaneous expression of both studied *TMPRSS2-ERG* isoforms. The detection rate in cancerous samples was also higher, 66 %, in the previous study, which could potentially be due to the sampling protocol which was more laborious and time-consuming in this study, potentially leading to RNA degradation and thus lower detection rates.

The tissue regions that were positively stained in ERG IHC matched the locations of carcinoma lesions, and either contained detectable *TMPRSS2-ERG* mRNA or were located adjacent to samples that did. However, all *TMPRSS2-ERG* mRNA positive areas did not stain positively for ERG even within a specific prostate, suggesting that the mRNA assays can reveal additional suspicious areas compared to IHC methods alone. This could be due to the IHC-negative areas not producing such amounts or forms of ERG protein that the antibody recognizes, even though previous reports of it have demonstrated correct identification of all the samples with the *TMPRSS2-ERG* rearrangement [15]. The rearrangement variant type did not seem to play a role in identifying ERG protein expression.

*PCA3* is a non-coding gene that does not produce a functional protein, so its expression was studied only on the mRNA level. *PCA3* is reported to be 10–100-fold overexpressed in prostate tumours [16, 17], but in our previous studies, we have seen values of this magnitude only when carcinomas were compared to prostates without clinical prostate cancer [9] and a 5.8-fold increase of *PCA3* expression in tumours was observed when they were compared to benign areas of cancerous prostates [8]. In this cross-section study, we detected a three times higher expression in the carcinoma samples than in the histologically benign areas next to malignant tumours or at locations further away. It would therefore seem that the increase in *PCA3* mRNA expression is not limited to the carcinoma foci in a cancerous prostate, but rather that there is a more global field change.

The set-up of this study is admittedly of a preliminary and experimental nature and due to the small size of the cohort, the conclusions can only be suggestive. The fact that only approximately half of prostate cancer cases are *TMPRSS2-ERG* positive [18] contributed to the even smaller number of *TMPRSS2-ERG* positive cases in our study. Additionally, we only studied one section of each prostate here in order to ensure the reliability of the routine pathological diagnostic procedure. This, however, could limit the conclusions drawn considering that prostate cancer, as also was seen in these specimens, is often a multifocal disease. The neoadjuvant therapy administered to three of the patients could also have had an effect on the results. However, despite these limitations, we find the set-up of this study novel and of interest. Clark and colleagues conducted a similar type of study using prostate cross-section slices and obtaining samples from cancerous and nonmalignant areas of the prostate, deemed as such based on the morphology of the slices above and below [5]. They, however, only looked at two matched samples from each prostatectomy specimen instead of the more systematic approach adopted here, which comprised a systematic and quantitative examination of mRNA levels throughout the tissue slice.

Utilizing these findings for diagnostic purposes would entail qRT-PCR assays performed on biopsies or tissue material obtained from transurethral resection of the prostate (TURP), where a finding of *TMPRSS2-ERG* mRNAs or high *PCA3* expression would indicate an increased risk of having or developing prostate cancer. While the routine formalin fixation may hamper the use of qRT-PCR assays, the recently introduced, alternative non-crosslinking fixatives such as PAXgene (Preanalytix) could be used for such purposes [19]. To our knowledge, there have been no reports of the fusion gene detected in tissues of men without any prostatic disorders, which supports the potential to use the fusion gene for risk analyses. However, there have been findings of *TMPRSS2-ERG* in the tissue of 6–8 % men with BPH but without history or suspicion of prostate cancer [5, 7]. This is suggested to be due to the fusion gene being a sign of early changes in the gland, but not always leading to malignancy, and naturally means that a positive *TMPRSS2-ERG* mRNA test result does not require the presence of a current prostate carcinoma.

## Conclusions

Our systematic study, despite its highly preliminary nature, shows that even though it is rare, it is possible to detect *TMPRSS2-ERG* transcripts in cancerous prostates in areas other than the carcinoma regions. If validated with larger cohorts and biopsy or TURP material, this could bring a new additive to assessment of risk of prostate cancer especially in men with negative biopsies.

## Abbreviations

BPH: Benign prostatic hyperplasia
cDNA: Complementary DNA
ERG: ETV-related gene
FFPE: Formalin-fixed, paraffin-embedded
HB: Histologically benign
HBAC: Histologically benign tissue immediately adjacent to a carcinoma/PIN sample
HE: Hematoxylin-eosin
IHC: Immunohistochemistry
KLK3: Kallikrein-related peptidase 3
mRNA: Messenger RNA
PCA3: Prostate cancer antigen 3
PCR: Polymerase chain reaction
PIN: Prostatic intraepithelial neoplasia
PSA: Prostate specific antigen
qRT-PCR: quantitative reverse-transcription PCR
TMPRSS2: Transmembrane protease serine 2
TURP: Transurethral resection of the prostate
